# STEP–organizing a major project to tackle significant uncertainty

**DOI:** 10.1098/rsta.2023.0402

**Published:** 2024-10-09

**Authors:** P. Methven

**Affiliations:** ^1^ UK Industrial Fusion Solutions Ltd., UK Atomic Energy Authority, Culham Campus, Abingdon, Oxon OX14 3DB, UK

**Keywords:** major projects, uncertainty, risk, organization design, strategy, empowerment

## Abstract

This article describes why Spherical Tokamak for Energy Production (STEP) has been launched, what it aims to achieve (benefits) and, principally, how the whole programme will be delivered (strategy). The article draws on the work of major project delivery and organization design (OD) and applies this to the context of STEP, which is dominated by significant uncertainty in all dimensions (technical, financial, commercial and programmatic), where there is embryonic delivery capability, but where there are also global-scale opportunities. This leads to an approach based on securing and organizing the correct capability from both public and private sectors to work in a collaborative arrangement with a single purpose and, critically, in an operating model designed to manage uncertainty and emerging risks and to exploit opportunities. Placing adaptability at the core of the OD, particularly the ability to deliver emergent strategy through guided empowerment in pursuit of an ambitious aim, is a further development beyond much of the current thinking in major projects. The article concludes with an appendix that translates that programme approach into principles for managing the engineering design work.

This article is part of the theme issue ‘Delivering Fusion Energy – The Spherical Tokamak for Energy Production (STEP)’.

## 1. The global context–need meets opportunity

The scale of the climate crisis [1] and the need for additional energy solutions to achieve sustainable net-zero carbon, especially as population rises [2] and per capita demand grows, has been coincident with technological advances in fusion energy. The UK is a recognized leader in fusion energy research, in large part owing to its hosting of the Joint European Torus, which has fostered a more integrated understanding of fusion plants as well as excellence in specific enabling disciplines such as plasma physics. Integrated experience has grown further with work on the Spherical Tokamak concept, which aims to reduce cost through smaller machine size for equivalent power levels compared with more standard aspect ratio tokamaks. The confluence of need and technical progress has led the to UK government forming a strategy to seize the resulting opportunity and translate a current research leadership position into one of commercial leadership. The UK Fusion Strategy [3] sets two clear objectives:

*‘For the UK to demonstrate the commercial viability of fusion by building a prototype fusion power plant in the UK that delivers net energy’.*

*‘For the UK to build a world-leading fusion industry that supports different fusion technologies and is capable of exporting fusion technology in subsequent decades’.*

Despite the totemic nature of the first objective, the second is ultimately more important as industrial capability is the route to sustainable economic returns that justify the investment. Spherical Tokamak for Energy Production (STEP) is the programme response to the first objective and a significant part of the means of delivering the second. Through the endeavour of designing and delivering a prototype power plant that is proportionally smaller and therefore cheaper than potential alternatives, STEP aims to develop industrial capability and deliver multiple wider benefits, described later. This marks a significant shift from previous decades of fusion research that have been dominated by the public sector [4]. The strategy also drives critical enablers such as regulation, where the UK is the first country to establish a specific regulatory framework for fusion energy separate from the approach to fission energy [5].

## 2. The case for public involvement

### (a) The nature of STEP

Notwithstanding the potential scale of the eventual economic benefits, the delivery context for STEP is one of great technical uncertainty, large scale (an infrastructure build of typical giga Watt fission plant scale), high programme complexity, long timescale, significant capital cost and embryonic supply chain capability (a function of the novelty of the technology). Unlike the idealized norm in infrastructure projects, it is not possible to fix requirements at the start and reduce change to the minimum: schedule and costs are extremely hard to estimate (affecting stakeholder confidence and funding models); linear (waterfall) management approaches are insufficient (even counter-productive) as many design paths will need to be stopped or altered; measurement and payment techniques such as earned value management become impossible to apply at the overall level as there is value in pursuing ultimately non-viable paths.

Inherent uncertainty and risk are compounded by concurrency. Concurrency is often present in major programmes and is equally often highlighted in post-project analysis as a root cause of failure; many project review recommendations read along the lines of ‘don’t start building until the design is fixed’. This is often hard to achieve in practice as, in anything of significant scale and complexity, progress is essential on some elements before others are fully ready. Fixed constraints such as planning windows, material availability or installation times for large items are typical drivers. For anything other than a repeat build (and those are rare in major projects), some aspects of design will not be ready until well after others and no amount of schedule management will change that. The question is how to manage concurrency rather than seeking to eliminate it.

These features, in aggregate viewed as the level of investment risk, help in part to explain why there is a significant role for the public sector, not just in providing science capability that is not yet deep and broad enough in industry, but in backing development work; however, there is equally a case for private sector involvement. While uncertainty and risk are generally viewed as negative, the corollary is business opportunity and so STEP must be able to identify and exploit opportunities, in part to deliver benefits but also because realising opportunities itself reduces the aggregate effect of risks.

### (b) Public sector stimulus

Significant public sector investment in early stage technology is controversial in some quarters though, as Mazzucatto [6] points out, is more common than often understood. The basis of what today are successful and purely commercial ecosystems (e.g. Silicon Valley) is often prior public sector investment that builds capability and gives rise to core technologies from which marketable products can later be developed at acceptable levels of commercial risk. In most cases, this progression arises over time and can appear serendipitous.

What is less common, at least more recently, and may be intrinsically more challenging, is large-scale capital investment in a publicly led programme that aims to stimulate commercial capability in the same sector. Examples tend to be historic and many have their roots in Defence where there is a national imperative, the need for initial state control and no direct private sector need for the specific product capability. A prime example is the US (leading to the UK) Naval Nuclear Programme, which also resulted in the US civil nuclear programme. A more recent example is the development of the Oxford-AstraZeneca COVID-19 vaccine. While UK policy backed a number of vaccine providers as a health risk mitigation, the big success story was the AstraZeneca vaccine where existing and part publicly backed capability in the Jenner Institute, which had the basis of a solution stemming from years of research, was coupled with industrial capability to generate a vaccine that was far more widely deployable and cheaper than alternatives. Many broader examples of public stimulus exist, including in energy. The UK has been a world leader in both the development and deployment of offshore wind energy with the renewables obligation on providers and, more recently, the ability to use contracts for difference driving growth [7].

### (c) Fusion as a mission-driven programme

Fusion energy represents one new example where public investment may be the only realistic approach to fully demonstrate the technology. Private investment in fusion has grown rapidly but, so far, those investments have not been sufficient to deliver a full-scale commercial prototype. That is largely because the capital cost will be multiple billions of pounds and as there is no realistic route to return on investment from power generation from a prototype alone; plant availability and energy generation levels from a first prototype/demonstrator will be low. As the reality dawns of the scale and complexity of viable fusion plant, many private fusion companies are combining their aim of whole plant design with a focus on core technologies that may have multiple and much nearer-term applications. Examples include firms focused on super-conducting magnets, which have multiple other uses [8].

Another issue, but one at the heart of STEP’s potential competitive advantage, is that solving all the key requirements needed for eventual commercial plants in one device probably requires a different design path to solving these requirements in isolation. In particular, designing for essential access and maintenance drives up size and is often in direct competition with features that help tritium breeding and/or net power generation. This complexity of design trades may not be as severe outside of magnetic confinement devices, but the experience in STEP over 4 years is precisely this—the integrated design cannot simply be an extrapolation of an intermediate design focused on a more limited set of requirements.

This market reality (scale of capital investment) and the technical realities that lie beneath it leave government as one of, if not the only, credible source of funding for initial full plant demonstration as governments have both access to significant funds over a long period and can justify and realize a wider range of benefits across the broader economy compared with a narrower revenue return basis of justification for commercial investors. True commercial plants of the future are unlikely to be, and arguably should not be, funded publicly but getting to the point where those plants can be privately funded is the most challenging stage of development.

## 3. Objectives and benefits

### (a) Whole programme objectives

The highest-level rationale for STEP is relatively straightforward to explain: de-risk a globally significant new technology with an enormous potential market through initial government support and develop an industry that will return that investment many times over. But more granular objectives are necessary both to justify the level of expenditure and to set the programme on a clear delivery footing, driving organization and focus. A set of whole programme objectives have been agreed for STEP; these are listed in table 1.

**Table 1.** STEP programme objectives.

Category	Top level objective
**Technical delivery**	**1–Demonstrate power plant characteristics** Design, build and operate a prototype fusion energy plant (the STEP prototype plant (SPP)) to demonstrate the key characteristics relevant to commercial power plants.
**Commercial pathway**	**2–Create an information baseline** Capture information through design, build and operations that will speed the delivery of commercial fusion at the lowest practicable cost and greatest benefit to the UK.
	**3–Develop a fusion supply chain** Through the delivery of the SPP, develop a supply chain capable of and committed to design and build of fusion energy plant.
**Value delivery**	**4–Deliver UK economic value** Deliver direct UK economic value stemming from delivery of the SPP, consistent with other objectives.
	**5–Deliver UK social value** Deliver UK social value stemming from delivery of the SPP, consistent with other objectives.
**Safety and environmental impact**	**6-Deliver safely** Reduce risks to workers, the general public and the environment from delivery and operation of the SPP to as low as reasonably practicable.
**Programme delivery**	**7–Schedule** Deliver SPP demonstrations and wider benefits as fast as reasonably practicable, underpinned by a robust whole programme schedule.
	**8–Cost** Deliver SPP demonstrations and wider benefits at the lowest practicable capital cost, underpinned by a robust whole programme cost estimate.

### (b) Benefits

In the UK public project model, measurable benefits are at the core of the ‘economic case’ within an overall business case. The highest-level benefit of STEP is a route to accessing a very large future market, known colloquially as the ‘size of the prize’. STEP funding can be seen as an option payment on this future opportunity. However, that opportunity is far distant and, from an investment calculation and policy point of view, speculative and highly discounted. To make an investment case that is at least marginally positive (mitigating downside) while still enabling an enormous opportunity, more tangible benefits must be identified and delivered.

### (c) Value now, not jam tomorrow

As well as ultimate benefits, it is important to realize benefits throughout the life of major projects and not simply at the end. From a government funding perspective, spending billions of pounds over decades with inherently uncertain final results is a difficult sell. Nearer-term benefits must be pursued to progressively mitigate that risk and to sustain support. Other stakeholders also need to see nearer-term gains: for example, local residents subject to years of construction transport disruption will want more immediate benefits. It is not always possible to fully achieve net near-term benefits but ignoring the need risks losing critical support as, in long-term endeavours, costs are generally borne by one generation and benefits realized by the next.

### (d) Wider benefits

Major projects are, by definition, transformational change endeavours: they seek to make a large intervention to change an existing paradigm. Benefits may be more or less direct with indirect benefits often greater but harder to realize as they are only enabled by the project and not under direct control, requiring other dependencies to come to fruition. For example, STEP plant build and operation will directly generate a significant number of jobs in the West Burton region where the plant will be built but should enable a far greater number by stimulating a business park/technology hub where a range of businesses located and where that net growth opportunity drives transport and housing improvements which in turn attracts more regional investment. STEP cannot directly manage all of those wider benefits but must work with local stakeholders deliver them.

## 4. Major project delivery

While there is increasing consensus in the literature and among practitioners on some of the core principles essential for leading complex major projects, much of that is centred on infrastructure projects where scale is significant and stakeholder interactions complex but where there is a generally understood solution. The nature of STEP as described in §2a requires an approach that goes beyond even more recent major project thinking, particularly on how to organize projects to embrace and manage uncertainty rather than seek to reduce it and accommodate it within better estimates. There may be useful parallels in other diverse fields subject to high uncertainty and opportunity such as transformational business change and even military operations. Four key themes in major project leadership are now examined that must form the basis of the approach in STEP, but further development beyond these principles is necessary and will be discussed in the subsequent section on strategy.

### (a) Quantifying and provisioning for uncertainty and risk

Flyvbjerg *et al*. [9] have shown the propensity for under-estimation of project schedule and cost and over-estimation of benefits. Whether the drivers are deliberate or not (Flyvbjerg’s ‘delusion and deception’), for a complex endeavour, it is not possible to fully capture all uncertainties and risks and, perhaps most importantly, the way those interact to drive a final outcome. To have certainty of final outcome would require precision on every activity over decades and of all possible risks and their interactions. Techniques such as Monte-Carlo analysis attempt to compensate by running multiple iterations of a project’s risk network but, even with use of correlation factors, are prone to generating narrow ranges owing to the treatment of risks as discrete when many are linked and can have partially common causes. To establish a more credible envelope Flyvbjerg *et al*. [9] advocate the use of the ‘outside view’ and the technique of Reference Class Forecasting.

This understanding is now permeating public sector advice. For example, the very sound recommendation from a recent UK Public Accounts Committee report [10] states ‘*Programmes should make greater use of cost and schedule ranges to reflect the high degree of uncertainty at early stages, with the expectation that these ranges would narrow as the programme is developed further.’* Another report on transport projects [11] states ‘*Avoid setting a committed in-service date before there is positive evidence that it is realistically achievable. Caveat dates as provisional and use a range showing the best case and worse case dates.’* The consequence that there is not a single number (date or cost) and indeed may not be until there is sufficient understanding is extremely challenging to manage and pressure from stakeholders can become intense, often defaulting to seeking certainty through applying greater central control. Pressures can also combine with internal project over-optimism leading to simplistic and un-caveated narratives and unrealistic estimates. Moreover, the firm bias of people to attribute more weight to and react more strongly to bad news [12] exacerbates the challenge of over-promise whereas it should lead to a lowering of expectations.

Notwithstanding growing understanding, policy has not yet adapted sufficiently by requiring, at some level of management, a significant provision for true contingency—a quantum of schedule and budget to cover for what is not and cannot be known in detail. Without that, if every unforeseen event outside of the initial estimate requires an uplift in budget, costs will only ever rise, confidence will erode, imposed constraints and scope changes will apply and benefits will be lost. This is the narrative of an out-of-control project.

### (b) Ownership of risk

In major projects, it is necessary to consider the aggregate risk at the programme level as distinct from the risks of discrete activities or subordinate projects; the former is significantly greater than the sum of the latter. The overall risk of delivering a first fusion energy plant is very large, hard to quantity and, importantly, beyond the realistic risk-bearing capacity and appetite of all commercial actors. Conversely, the risk of delivering some discrete and specifiable elements of the overall plan, e.g. standard office buildings, is quantifiable and therefore capable of commercial ownership through risk pricing models. The Heathrow Terminal 5 project analysed behaviours likely to result from attempts to transfer overall project risk in the traditional way of the UK construction sector. The analysis, involving game theory, showed the negative effects that would result (frequent contract variation claims) and led to the conclusion that the client had to explicitly own overall risk [13].

### (c) Securing the capability–a collaborative approach

The span of capabilities needed in major projects requires multiple organizations to work together. In STEP, much of the capability in fusion exists in the public sector, whereas the capability to develop large-scale engineered products and facilities resides in the private sector. The complex nature of the challenge and the distance from any clear specification means that the nature of working between participants must be collaborative in pursuit of a common aim [4]. This is simple to state but, as relationships are generally implemented through contractual mechanisms, challenging in practice. Moreover, the capability needed will evolve as the project moves through phases and as the solution matures.

### (d) Organizing the capability–project as organization

In practice, major projects are not well matched to traditional definitions of projects such as ‘a unique, transient endeavour undertaken to bring about change and to achieve planned objectives’ [14]. One aspect driven by time and scale is the organizational construct required to deliver effectively. Where an organization designed for one purpose finds the need to deliver a project of scale and complexity, its operating model is unlikely to be optimized for that purpose. Bespoke project processes are not the same as routine business processes, the skills and experience of the people will differ, and the capabilities required will probably come from multiple individual organizations. Major projects need to build a dedicated organization matched to the task. Van Donk and Molloy [15] and earlier work by Van der Merwe [16] addressed the need to integrate strategy, structure and process specifically in a project management context. Most organization design (OD) models stress the need for a coherent and complementary approach to the elements and to avoid a myopic focus on structure as the dominant (often sole) feature of design; the Waterman *et al*. ‘7s’ model [17] is a well-known example. Beyond the now broadly accepted thinking that major projects at least merit clear OD, two other factors are worth considering but less discussed.

**Figure 1.**
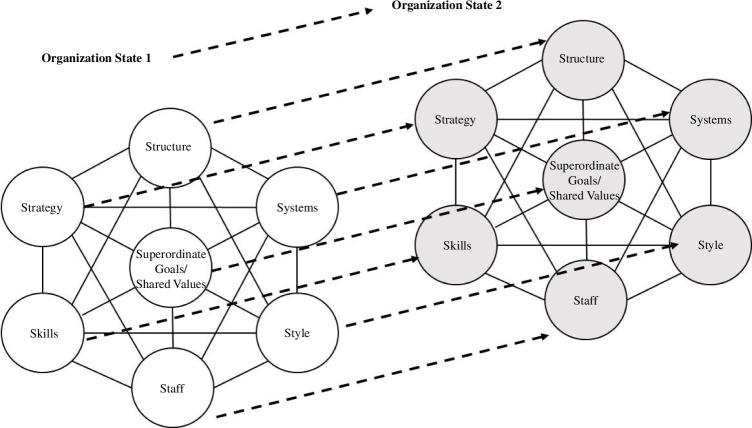
Evolving OD through project phases, adapted from Waterman *et al*. [17].

First, projects themselves are necessarily different through each major phase. For example, the skills and approach needed in early stage design differs markedly from those needed in commissioning. Usefully, the 7s model was developed with organizational change in mind, a key point being that the balance of each element must be correct for the intended future state and transitions must consider all elements. Figure 1 shows the 7s model evolving between states in the project lifecycle, where each state must be balanced and self-consistent between the elements.

**Figure 2.**
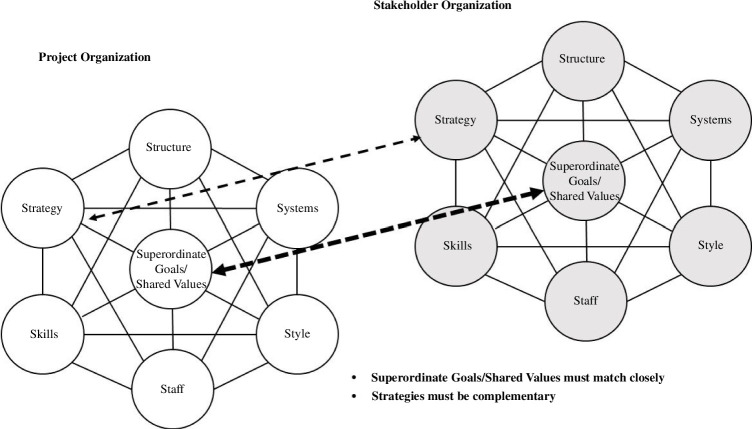
OD mapping across stakeholders, adapted from Waterman *et al*. [17].

Second, the interaction between a major project and its operating environment (the set of actors and conditions that shape and constrain the project) is so fundamental to success that OD may also be necessary for elements of that wider environment (such as for the Project Sponsor organization) or, at least, understanding the OD of key stakeholders in practice will enable potential dissonance between project and environment to be better understood and managed. This is represented in figure 2, showing a central project OD interacting with a stakeholder OD, which will have different balances of all seven elements; ODs cannot be the same but for effective joint working must be complementary. Significant differences in shared values and strategies will lead to failed relationships. This is especially true for commercial partners where despite significantly different ODs between client and supplier there must be alignment on at least some core values, and a clear unifying purpose.

## 5. Delivery in practice–strategy and guided empowerment

In simpler projects it is more (though not wholly) possible to rely on delivery plans as the linkage between intent and action, but the more complex and uncertain the endeavour the more the need for an adaptable approach at the highest level while more bounded subelements can have defined plans. This is the domain of strategy, which is a component of OD. Rumelt [18] argues that the key dimensions of a strategy are an analysis (reflecting that strategy depends on specific context), a guiding policy and a set of key actions. The context for STEP, the analysis in Rumelt’s terms, has been described above with the key elements of embryonic and uncertain technology and industrial capability, scale, complexity, public funding and immense opportunity.

### (a) Factors in STEP strategy

A core strategy consistent with that context requires embodiment of some dimensions of major project thinking, in particular: ensuring a realistic envelope with adequate contingency; client owning overall risk; securing collaborative capability across public and private sectors; and, most importantly, the need to design an organization matched to the context and challenge. Beyond much current project literature, the organization needs to be able to evolve as the project moves through phases and must to be able to mesh with or at least influence critical stakeholders. In the STEP case, there are three further considerations.

First, the project organization will grow from within an existing organization and must be different in many dimensions but also complementary with the design of its parent. To that starting point will be added private sector capability, enabled by contracts, and that collective capability will need to blend into a cohesive team with a single purpose. Second, while industry must be part of the overall model in order to deliver a plant of this complexity, it is also an overarching objective to develop industry capability through the work, and this requires an even more integrated and relationship-based model than is used in most complex major projects. Third, the level of uncertainty in STEP is far beyond almost any other major infrastructure or technical project and the ability to manage that uncertainty internally and with stakeholders is beyond much major project thinking rooted in infrastructure. Even the route to the final outcome is uncertain as knowledge gathered at each stage will drive progressive adaptation of the plan. Managing this may benefit from examining the approach to more intrinsically uncertain endeavours such as social change programmes or military operations.

### (b) STEP strategy and themes

With all these facets taken together, the basis of the strategy for STEP can be set out as:

*Secure and organize the right mix of public and industry capability into an integrated delivery team under clear client leadership, focused on delivering a cost-effective prototype fusion plant at a pace that secures UK leadership in fusion and delivers consistent value.*

Five key themes are core to this:

***Build industrial capability.** Build and continuously develop industrial fusion plant delivery capability, at all levels of the supply chain and where practicable in the UK, through design, build and operation of a commercially relevant prototype fusion energy plant*.

***Organize for success.*** *Organize and lead that collective public and private sector capability under UKIFS to manage significant inherent uncertainty and to exploit opportunities in technology, manufacture, regulation and investment*.

***Design and deliver for cost.*** *Design and deliver an integrated fusion energy prototype plant to be as low cost as practicable, with a trajectory to low-cost commercial plant, while accommodating intrinsic uncertainty in technology, build and operations*.

***Drive pace.*** *Develop the culture and systems to make decisions and deliver at pace to secure UK competitive advantage, support third-party investment and tackle climate change*.

***Deliver value consistently.*** *While driving for final outcomes and benefits, ensure tangible value is delivered at all stages of the programme, building stakeholder and investor confidence and reducing government risk*.

### (c) OD in practice

**Figure 3.**
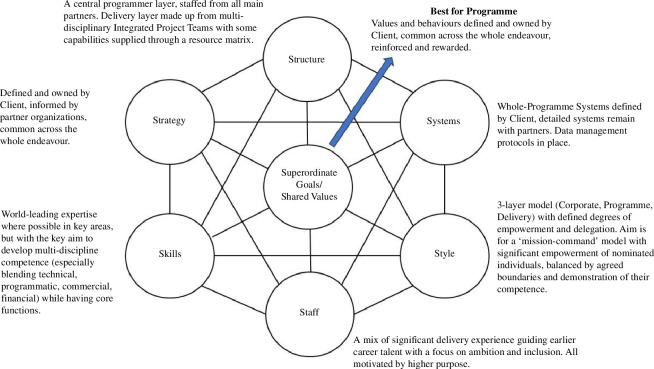
STEP OD basis.

**Figure 4.**
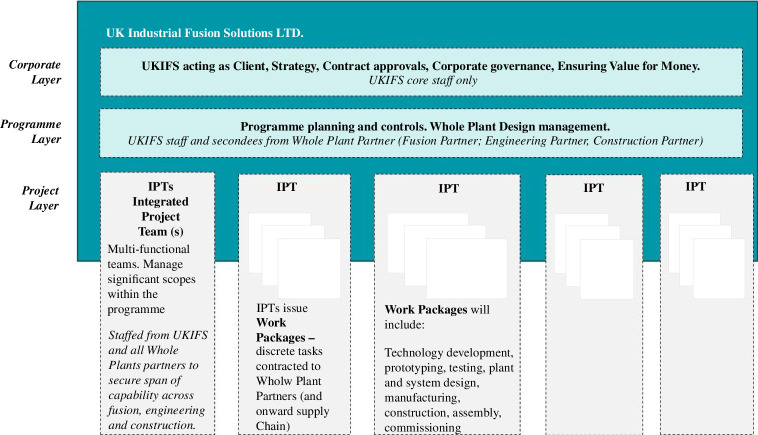
STEP target operating model, from Chapman *et al*. [4].

The strategy and its themes have been developed iteratively with an overall operating model to integrate partners with varying ODs. Figure 3 illustrates this for STEP using the 7s model, and figure 4 shows the intended as a more traditional organizational diagram.

The model is led by a new bespoke major project client organization, UK Industrial Fusion Solutions Ltd. (UKIFS), incubated within by UKAEA. Commercial partners are contracted on the basis of the capability they provide, their strategic alignment and their values and behaviours, and are highly integrated into UKIFS. Core UKIFS staff operate the corporate layer, having responsibility for strategy and overall governance. Partner staff are seconded into the client organization at the programme layer to provide expertise to shape and plan the work. The delivery layer comprises multi-disciplinary integrated project teams (IPTs) teams focused on the main areas of the plant and programme, organized by product breakdown. There is a ‘one-team’ approach across all partners, based on the shared value of ‘best for programme’. Commercial arrangements are of an alliance nature with upside incentivization for collective performance, and overall risk owned by the client. The model is inherently adaptable, allowing IPTs to evolve and grow in scale and scope as the overall project evolves, with a lean central management layer maintaining alignment. Umbrella contracts allow successive work packages to be developed and placed with the partners, and then onwards to the supply chain, as the next stages of work become clearer.

The bulk of the technical effort resides in the IPTs, staffed from across the alliance partners, which will take responsibility for significant scopes of systems and subsystems and, critically, the sourcing and management of the onward supply chain needed to deliver those systems. This seeks to match markets to technology. The whole plant design team, responsible for integration of all systems and transversal aspects such as requirements management, safety, environment, maintainability and availability work in the programme layer, with a very close relationship to the programme management and controls team.

This design is specific to the context and strategy of STEP and will differ from the OD of other fusion endeavours. For example, privately backed start-ups that aspire to deliver full-scale plant will follow a very different organizational evolution driven much more by the demands of discrete investment rounds that likely focus on more specific proximate milestones in key technologies. Other national programmes may differ depending on procurement rules or whether they seek to stimulate a market as well as demonstrating technology. The OD may ultimately prove part of STEP’s competitive advantage and, if it does, this will probably be because of the explicit focus on agility compared with more traditional project design, and because the capacity to move from concept to large-scale manufacture has been embedded early. Integrating industrial capacity early may initially slow delivery and will seem less nimble than private approaches but seeks to prepare for the subsequent (harder) stages of delivery where time is often lost. Nevertheless, one of the things STEP must achieve, including across its key stakeholders, is a measure of entrepreneurial zeal within a public sector and major programme context, not an easy task.

### (d) Deploying the strategy–guided empowerment

With context understood, and a core strategy and operating model defined, an overall plan of critical high-level actions can be developed. However, simply declaring a strategy is not enough—the approach must also consider how strategy is deployed. Arguably, this is a core element of OD and is most closely represented in the 7s model by the element of ‘style’. In literature on lean production [19], itself a transformational change in business, this challenge is termed ‘Hoshin Kanri’—policy deployment. Some may refer to this as ‘ways of working’ or, more straightforwardly, how to get people at all levels, and in this case from multiple parent organizations, to follow the strategy.

**Figure 5.**
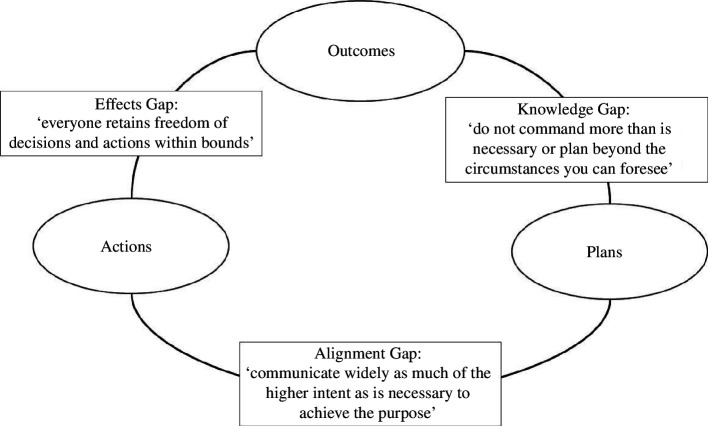
How style operates in STEP, adapted from Bungay [20].

Project management literature has much less to say about this, but the question has dogged military leaders for longer than it has business leaders. Bungay [20] has attempted to codify what has been the basis of at least western military leadership and strategy deployment and which a number of successful businesses have embodied. The military concept is termed Mission Command which, despite the title, is a devolved (not centralized) approach where lower levels understand deeply the vision and intent at senior levels and are empowered to set their own objectives to achieve that higher level intent, within clear but often wide boundaries. The environment is supportive and includes back-checking, and, importantly, empowerment is not automatic but depends on demonstrated competence. An alternative title would be ‘guided empowerment’. The approach is designed specifically to cope with large uncertainties, risks and unforeseeable exogenous changes while maintaining momentum towards a clear goal. Without this specific ability, the overall design represented in figure 3 would be unbalanced. Insufficient guidance and alignment would be chaotic and poorly performing, but excessive control (which is illusory) would stifle innovation and result in an unmanageable bureaucracy of change control. Figure 5, adapted from Bungay [20], describes the approach intended for the style element of the STEP model.

## 6. Conclusions

STEP is part of the UK’s strategy to take a commercial lead in the new technology of fusion energy, with the objective of realizing significant economic benefits should fusion be in demand to address foreseeable and otherwise unfilled gaps in future energy demand. It is a major project with an unusually high degree of uncertainty and a low starting point in terms of delivery capability, which also provides the basis of the associated economic opportunity. As with all major projects, delivery requires a bespoke operating model, in this case integrating public and private sectors both to secure the capability to deliver the prototype and to develop industrial capability as the source of sustained economic value. The specific OD, developed in concert with the programme strategy, embodies lessons from major project literature and practice but must go further, in particular in the dimension of style. Here, practice evolved over centuries from the intrinsically uncertain and emergent sphere of military operations, more recently seen in many businesses, offers the approach of mission command or guided empowerment. The STEP model integrates this into its operating model as a direct response to uncertainty.

## Data Availability

This article has no additional data.

## Appendix A. Realizing programme strategy in the technical domain through design principles

While the core programme strategy at this stage is development and integration of adaptive capability, much of that capability will be applied to the technical challenge and requires more specific guidance.

### (a) Iterative design

Whilst the overall programme will proceed in an apparently linear progression of design, manufacture, assembly, test, commissioning and operations, the inherently exploratory nature means there will be significant iterative development within each major phase and across phase boundaries. Even in operations, the early stages are not simply about proving the design but about experimentation and validating very large physics assumptions, some of which may drive very late and major changes. The early design work is a particular case where there will be multiple iterations at each system and subsystem and where those need to be brought back frequently into a coherent whole plant design baseline to ensure coherence. Moreover, as the whole problem is one of trading performance and programme parameters, requirements must evolve in parallel with the solution rather than being set arbitrarily at the start (for more details see [21,22]).

### (b) Discrete and aggregate risk

STEP has an intrinsically high-risk appetite. In key areas, significant levels of technology development risk may be accepted where there is a critical performance, schedule and/or cost benefit. To accommodate higher-risk choices in those areas, other areas of the design (and wider programme) should seek deliberately lower-risk approaches to manage the aggregate level of risk (for more detail see [21]).

### (c) Showstoppers, and relative versus absolute decisions

Design choices will initially be focused on ruling out what does not work and only later will be between viable alternatives with differing benefits and risks. Almost all design decisions are relative rather than absolute and each discrete choice may be far from ideal and carry significant risk but need only be judged as better than viable alternatives; only the final overall concept needs to be judged as acceptable or not against programme objectives (for more detail see [21]).

### (d) Practical limits and constraints

While some initial options are likely to be non-viable, requiring current or foreseeable physical limits to be exceeded, there will be a need to challenge constraints and limits in some areas. Understanding flexibility in real and perceived constraints is vital.

### (e) Margin and resilience for uncertainty

Given intrinsic high uncertainty, in particular of the plasma scenario through all phases (ramp up, ‘stable’ operations, ramp down and faulted conditions) and of component performance over a lifecycle of changes through neutron damage, the design must be sufficiently resilient (carry enough margin) for that span of uncertainty to operate away from more challenging limits (for more detail see [21]).

### (f) Smallest and cheapest consistent with resilience

The design should be the smallest possible solution consistent with those margins. Technical solutions often experience increases in size through the detailed design phase and so commencing with the smallest solution is sensible in terms of managing cost (for more detail see [23]).

### (g) Complexity and quality

A key driver of cost and low availability is reduced complexity. As plant availability will be driven to a significant degree by the end-to-end reliability of key systems, the number of systems and components must be limited and have high-engineered quality. This will drive reduced lifecycle costs and, probably, reduced overnight cost. To reduce overall complexity, critical individual elements of the design will probably require elegant and novel engineering solutions, and this is preferable to the illusory benefit of multiple inter-dependent but ultimately unproven systems (for more detail see [21]).

### (h) Maintainability focus

While maintainability is an objective, it is also a design principle. Every specific piece of design should aim to address maintainability rather than maintainability being simply the default outcome of all decisions (for more detail see [24]).

### (i) Plant lifetime

Ultimate commercial plant will need significant overall and individual component lifetime to deliver availability and to limit waste volumes. For the first prototype, however, the reality of the limited palette of materials available and the paucity of data on performance in a high-energy neutron flux means that seeking an arbitrary lifetime should not dominate choices unduly. Where lifetime and/or the balance between overnight and lifetime costs is clear and a realistic discriminator, that is welcome but the design must first work and only then should work for longer (for more detail see [21]).

### (j) Benign safety

A benign plant with a high tolerance to faults is far preferable to a multiplicity of back-up systems that increase complexity and reduce reliability. The design should primarily focus on functionality and, where possible, the inherent capability of duty systems should also provide safety functions without skewing the design or increasing complexity. Design should minimize the need for additional and separate safety systems. In evaluating choices, the ALARP (As Low As Reasonably Practicable - the basis of ensuring safety in accordance with the UK’s Health and Safety at Work Act 1974) principle is paramount to reducing risks. The design must be viable and practicable first, and then the inherent risks must be reduced to reasonable levels. Finally, there is a significant distinction between safety and asset protection; there may be a significant higher acceptance of asset damage than of risks to workers and public (for more detail see [25]).

### (k) Safety despite uncertainty

There is no extensive evidence base of fusion component or system performance robust enough to underpin a modern standards safety case in the way that has become common in civil fission. As a result, while the plant should be engineered well, explicit claims made within a safety case should be limited to robust barriers that constrain consequence instead of, as is more normal, preventative features earlier in fault sequences (for more detail see [25]).

### (l) Decide now versus decide later

If data and analysis to support a decision will not be available or alter materially by the last responsible moment for that decision, the decision should be made earlier to reduce overall variability.
